# A mixed-methods protocol to develop and validate a stewardship maturity matrix for human genomic data in the cloud

**DOI:** 10.3389/fgene.2022.876869

**Published:** 2022-10-14

**Authors:** Vasiliki Rahimzadeh, Ge Peng, Mildred Cho

**Affiliations:** ^1^ Baylor College of Medicine, One Baylor Plaza, Houston, TX, (United States); ^2^ Earth System Science Center/NASA MSFC IMPACT, The University of Alabama in Huntsville, Huntsville, AL, (United States); ^3^ Stanford Center for Biomedical Ethics, Stanford University, Stanford, CA, (United States)

**Keywords:** stewardship, human genomics, ELSI (ethical, legal, and social implications), data governance, cloud, Delphi

## Abstract

This article describes a mixed-methods protocol to develop and test the implementation of a stewardship maturity matrix (SMM) for repositories which govern access to human genomic data in the cloud. It is anticipated that the cloud will host most human genomic and related health datasets generated as part of publicly funded research in the coming years. However, repository managers lack practical tools for identifying what stewardship outcomes matter most to key stakeholders as well as how to track progress on their stewardship goals over time. In this article we describe a protocol that combines Delphi survey methods with SMM modeling first introduced in the earth and planetary sciences to develop a stewardship impact assessment tool for repositories that manage access to human genomic data. We discuss the strengths and limitations of this mixed-methods design and offer points to consider for wrangling both quantitative and qualitative data to enhance rigor and representativeness. We conclude with how the empirical methods bridged in this protocol have potential to improve evaluation of data stewardship systems and better align them with diverse stakeholder values in genomic data science.

## 1 Introduction

Genomics is a data-intensive science requiring extensive research collaboration across institutions and international borders. Research institutions face mounting pressure co-locate secure access, use and exchange of data to drive innovation in genomics (Langmead and Nellore, 2018). In addition to decentralized and federated access models, national research agencies are heavily invested in cloud technologies to enable controlled data access (Stein et al., 2015). This migration to the cloud represents an important shift not only in how data repositories stand up their privacy and security infrastructures, but also in how repository managers steward the data resources generated by research supported through public funds (Grzesik et al., 2021). Genomic data are uniquely identifying not only for the individual about whom data specifically relate, but also for their biological relatives and communities (Song et al., 2022) in which they live and work. Sharing genomic data also comes with increased risk of re-identification. Recent studies have shown, for example, that individuals can be re-identified from aggregate datasets with few record linkages (Dwork et al., 2017). These properties affect how genomic and related data are collected, regulated, and shared.

We refer to data repositories in this article as entities which store, organize, validate, archive, preserve and distribute genomic and related health data submitted by the community related to particular system(s) in compliance with the FAIR (findable, accessible, reusable and interoperable) Data Principles (NIH, 2022a). At a minimum, data stewardship can refer to the institutional practices and policies meant to calibrate appropriate data protection with compliant data access and use. Data stewardship is thus integral to well-functioning data governance systems (Boeckhout et al., 2018) that requires practical frameworks for compliance as well as stakeholder-engaged research on values and priorities.

Yet while commitments to responsible stewardship are outlined in repository data sharing policies, and methods for evaluating stewardship impact have been proposed (Wilkinson et al., 2016), these are largely underdeveloped for cloud-native environments with few exceptions [see for example access policies for the research analysis platform of the United Kingdom Biobank (UK Biobank, 2022) and NIH Cloud Guidebook (NIH, 2022b)].

We lack empirical data, for example, on what stewardship outcomes matter most to key stakeholders and how we should measure them over time. Examples of stewardship outcomes could include concordance between consent permissions and data use restrictions, ethics review of proposed data uses, processing times for data access requests, and the number of successful data access requests among researchers working in low-and middle-income countries. According to its access procedures, for example, United Kingdom Biobank’s cloud services charges fees for tiered access as well as data storage and analysis of data. While reduced access options are available, it is unclear whether pay-for-access policies affect who can afford to conduct the research in the first place.

In this article we describe a mixed-methods study design to identify stewardship outcomes and develop assessment criteria for assessing them in cloud-native environments. We first discuss the unique properties of genomic data and the ethical, legal and social issues of migrating such data to the cloud. We then explain how current genomic data management and access challenges the ways that repositories practice responsible stewardship in these new computing environments. In response to these practical challenges, we describe how a modified Delphi together with stewardship maturity modeling can be used to develop, validate and test the implementation of a stewardship impact assessment tool for global repositories which host data in the cloud. Next, we discuss analytical approaches for wrangling both quantitative and qualitative data generated in the proposed study, raising points to consider for ensuring rigor and representativeness. We conclude with how adapting SMMs for tracking progress on data stewardship can advance a new research agenda for evidence-based stewardship in human genomics as computing capabilities evolve.

### 1.1 Cloud infrastructures and the need to store, analyze and share human genomic data at enterprise scale

New digital infrastructures powered by cloud technologies transform how researchers interact with, analyze, and share data at scale including in clinical areas such as cancer (Langmead and Nellore, 2018) (Lau et al., 2017) and rare disease (Zurek et al., 2021). Using cloud services as infrastructure to host the largescale genomic data collections—one of four distinct types of cloud service separate from software as service (SaaS), platform as service (PaaS) and serverless (O’Driscoll et al., 2013)—offers powerful advantages (Stein, 2010). These include simplifying management (Schatz et al., 2022), overcoming security risks associated with traditional copy and download, and making data available in organized, searchable formats which reduce time and resource burdens (Kudtarkar et al., 2010).

However unique features of these computing environments compel new ethical, legal and social questions about how to responsibly access and steward genomic data in the cloud (Carter, 2019) (Filippi and Vieira, 2014). For example, data protection laws are jurisdiction-specific while actual data users may be based all over the world. This complicates which data protections regulations should principally apply: those in the jurisdiction where the repository is based, where the user resides, or both? Many repositories purchase cloud services from commercial providers (e.g., Google, Amazon Web Services), raising some concerns about the dependence on third parties and potential for interference (Molnár-Gábor et al., 2017). As Philipps and colleagues argue, “service outages caused by technical problems, changes to the company”s terms of service or even sudden closure of the company could block researchers’ access to data at any time. Also, it is often unclear to what extent researchers using cloud services can ensure that their data are not disclosed to third parties, such as those conducting abusive state-level “surveillance” (Phillips et al., 2020).

While there is broad consensus on data stewardship principles outlined in frameworks such as FAIR, TRUST, and CARE (Table 1), their assessment has been computationally difficult to perform in practice (Anjaria, 2020). It has been shown how modeling a stewardship maturity matrix (SMM) can be effective at capturing the FAIRness of datasets and TRUSTworthiness of repositories in the earth and planetary sciences (Downs et al., 2015) (22). SMMs are often presented by a two dimensional array mapping n stewardship outcomes of interest onto various levels of organizational development (Peng et al., 2015): ad hoc, minimal, intermediate, advanced and optimal. A sample SMM is presented in Table 2. Across the rows of the matrix reflect “various facets of core stewardship functionality, (e.g., data management), while the columns describe typical behaviours representing increasing maturity in practices and capability against each aspect, ranging from a poorly-managed or no-capability state to an advanced, well-managed state” (23). Once developed, the SMM “can be used not only as a guide to users about the rigour of data stewardship practices, but also as a tool for monitoring and improving aspects of organizational performance in producing, managing, or servicing climate data” (Dunn et al., 2021).

**TABLE 1:** Data stewardship frameworks.

Stewardship framework	Stewardship focus	
FAIR (Wilkinson et al., 2016)	Findable, Accessible, Interoperable, Reusable,	Datasets
TRUST (Lin et al., 2020)	Trust, Respect, User-focused, Sustainability, Technology	Data repositories
CARE (Carroll et al., 2021)	Contribute, Attribute, Release, Empower	Data stakeholders (e.g. data users, creators, regulators, contributors)

**TABLE 2:** Template stewardship maturity matrix that charts n stewardship outcomes of interest onto five descriptive layers of organizational development.

	Outcome n	Outcome n + 1	Outcome n + 2
Ad hoc (not managed)	Ad hoc criteria for outcome 1	Ad hoc criteria for outcome 2	Ad hoc criteria for outcome 3
Minimal (limit-managed, not defined)	Minimal criteria for outcome 1	Minimal criteria for outcome 2	Minimal criteria for outcome 3
Intermediate (managed, defined, partially implemented)	Intermediate criteria for outcome 1	Intermediate criteria for outcome 2	Intermediate criteria for outcome 3
Advanced (well-managed, well-defined, fully implemented)	Advanced criteria for outcome 1	Advanced criteria for outcome 2	Advanced criteria for outcome 3
Optimal (measured, controlled, audited)	Optimal criteria for outcome 1	Optimal criteria for outcome 2	Optimal criteria for outcome 3

Several reasons justify exploring how SMMs can be adapted to study human genomic data stewardship outcomes. First, advances in human genomics, like earth and planetary sciences, depend on sharing high quality and well managed data resources. Second, large, publicly funded repositories are among the primary sources where researchers access the data they need to conduct rigorous genomics research. Therefore data access and release activities catalyzed by repositories makes them strategic focal points for assessing stewardship outcomes (Dunn et al., 2021).

## 2 Methods

**FIGURE 1:**
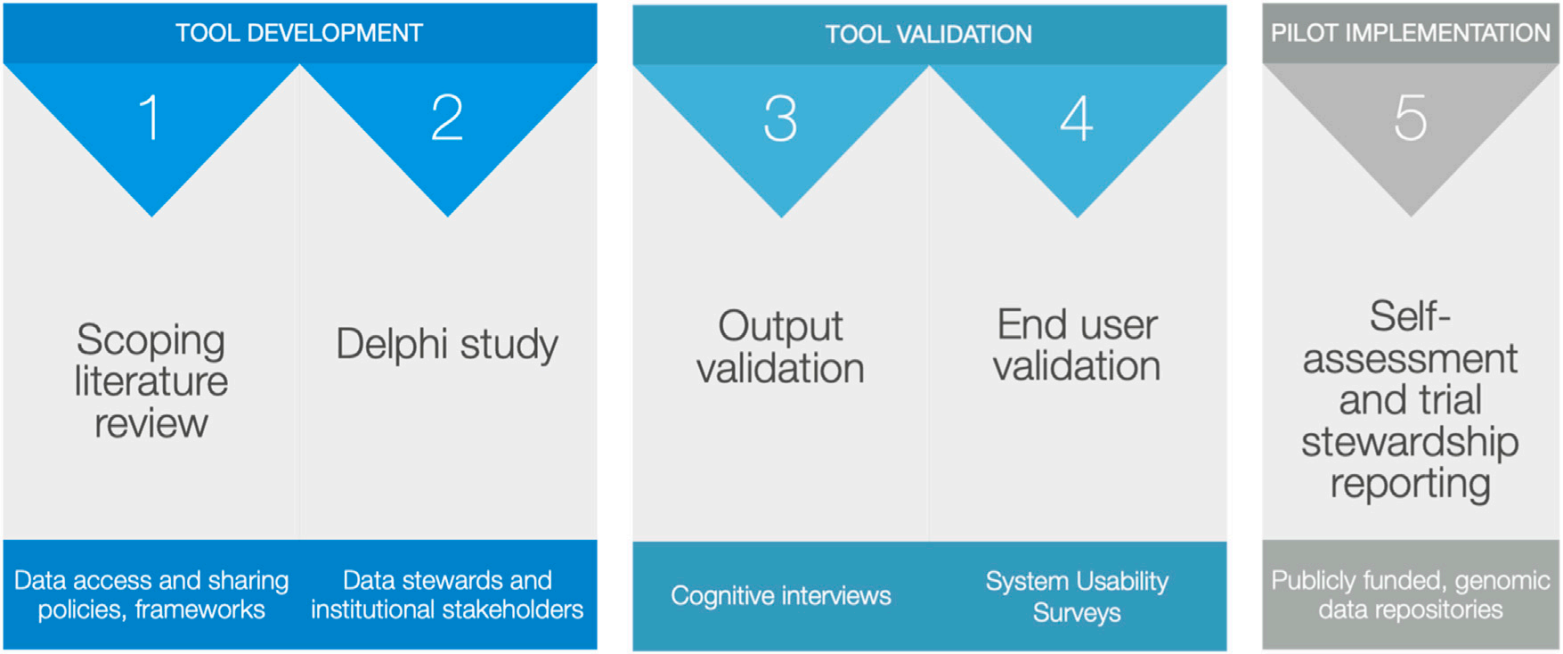
Step-by-step outline of a protocol to develop, validate and pilot the implementation of a stewardship maturity matrix tool to track progress on human genomic data stewarded in the cloud.

In the sections that follow, we provide methods and instructions for how to first develop (phase 1) validate (phase 2) and then test the implementation (phase 3) of a SMM for human genomic and related health data managed in the cloud. An overview of the protocol, as well as the specific materials and equipment used are provided in Figure 1 and Table 3, respectively. First, a scoping review of data sharing, management and access policies inform an initial core outcomes set for responsible data stewardship bespoke to cloud-native repositories. These core outcomes are then evaluated and further refined by actual repository managers, privacy officers and other institutional data stewards in a Delphi study. Institutional stakeholders engaged in the Delphi will also work to develop assessment criteria specific to each core outcome in a process that will result in a draft SMM. The SMM will be field tested with topic experts and piloted within repositories that currently host genomic data in the cloud.

**TABLE 3:** Materials and equipment used in the protocol organized by study phase.

Research phase	Materials and equipment used
Laptop computer, internet access	
Phase 1: Identifying core outcomes of genomic data stewardship	• Library services/access and librarian support
Phase 2. Developing the stewardship maturity matrix	• Online survey platform, with optional software applications specific to Delphi surveys (e.g. Welphi available at https://www.welphi.com/en/Home.html
	• Qualitative data analysis software (e.g. Dedoose, NVivo)
	• Quantitative data analysis programs (e.g. R, STATA)
Phase 3. Validation of the stewardship maturity matrix tool	• Video conferencing services
	• Qualitative data analysis software (e.g. Dedoose, NVivo)
	• Quantitative data analysis programs (e.g. R, STATA)

### 2.1 Phase 1: Identifying core outcomes of genomic data stewardship

The objective of Phase 1 is to inform a core outcomes set (COS) for genomic data stewarded in the cloud following a scoping literature review of data sharing, management and access policies (see for example Ethics and Governance Framework for the United Kingdom Biobank); published data stewardship frameworks, empirical studies, guidelines, and best practices. A detailed search strategy will be developed with guidance from a reference librarian, and which will include relevant search terms such as “genomic data,” “stewardship,” “cloud,” “infrastructure,” “data sharing,” “outcomes” among others to best capture existing stewardship measurements and approaches. An example search strategy is provided in the Supplementary Material S1.

### 2.2 Phase 2. Developing the stewardship maturity matrix

Findings from the literature review will inform an initial COS that will be refined in a three-round Delphi survey involving institutional data stewards, repository managers and other data access and privacy officers working at genomic data repositories globally.

Delphi methods are particularly well suited to refining COS and have been used in previous bioethics work to guide genomics policy (Stevens Smith et al., 2020). Delphi studies engage informed stakeholders through iterative rounds of structured communication and feedback (Banno et al., 2019). A Delphi facilitator collects panel responses, usually anonymously, and statistically aggregates and analyzes them (Rowe et al., 2001). The facilitator then provides summaries back to panelists who are invited to re-evaluate their position after considering responses from fellow panelists. This process is iterated across several rounds until reaching a pre-specified threshold indicating a consensus pattern.

The Delphi survey will enable panelists to evaluate each outcome for its relative importance and feasibility, suggest new outcomes and vote to eliminate those that are either infeasible to implement or unable to be measured in practice. In the final round of the Delphi, panelists will convene to develop assessment criteria specific to each core outcome and map these onto a two-dimensional array shown in Table 2.

#### 2.2.1 Phase 2 participant selection

Prospective panelists should represent institutional stakeholders with expertise in data management and data access review (e.g., data access committee members, privacy officers, managers) across repositories which currently use cloud services or plan to in the future. Panel membership is critical to the external validity of the resulting SMM. We will therefore carefully consider personal attributes such as relevant expertise, experience, availability, and representativeness to guide recruitment decisions using Table 4 as a guide. Published studies also reported that offering incentives improved panel retention and enhanced the quality of participation (Belton et al., 2019) without unduly pressuring participation. As is customary, we plan to compensate Delphi panelists using rates typical of professional consultation in their respective fields.

**TABLE 4:** Practical guidance for planning an expert Delphi panel.

Attribute	Questions to consider	Useful indicators	Protocol-specific guidance
Relevant expertise	o What professionals are involved in or implicated by the policy topic?	o Degree credentials	Professionals with relevant expertise could include
	o What industries are affected?	o Professional background and training	o Data stewards
	o What community groups are affected?	o Job description	o Data producers
		o Employer	o Data access committees
			o Repository managers
			o Data infrastructure designers
			o Software engineers
			o Cloud service providers
			o Policy and governance leads
Availability	o Do you have a pre-existing relationship with the prospective panelist or their professional community?	o Informational interview with prospective panelists	o Schedule interviews before/after work hours
	o Are there constraints on the panelists’ time?	o Publicly available contact information	o Compensate panelists for afterhours participation
	o Can they be contacted?		o Avoid participation during peak holiday months
	o Can they access communication channels?		
	o Are they willing to sustain their participation?		
Representativeness	o Is the demographic distribution of prospective panelists reflective of the stakeholder community?	o Published literature	o Leverage members in existing professional networks/societies (e.g. Global Alliance for Genomics)
	o What is the demographic distribution of panelists in terms of age, gender, profession, years of experience, race/ethnicity/religion	o Demographic reports	o Consider oversampling from underrepresented groups
		o Census data	o Conduct online search of active human genomic data repositories globally

#### 2.2.2 Phase 2 data collection

In Round 1 of the Delphi, we will capture panelists’ perspectives on the relative importance and feasibility of each core outcome (Sinha et al., 2011) and allow panelists the opportunity to contribute additional outcomes. We intend to pilot each round of surveys among a group of topic-naïve experts to ensure overall comprehension. To discourage ambivalent responses, we will adopt a three point Likert scale for rating exercises (Lange et al., 2020). Embedding free text responses in the survey will allow us to triangulate quantitative survey data with qualitative analysis of the rationales panelists provide for each core outcome. In Round 2 of the Delphi, panelists will re-rate outcomes that failed to reach consensus in Round 1 after reviewing the results and panel summaries. A summary report of survey results and qualitative rationales from Round 2 will be given to panelists prior to a 60 min virtual consensus workshop in Round 3. During the workshop, panelists will provide input on draft assessment criteria specific to core outcomes deemed to be essential after Rounds 1 and 2. We will use a progressive maturity scale—the capability maturity model integration™ (Carnegie Mellon University, 2001)—to match core outcomes with assessment criteria.

#### 2.2.3 Phase 2 data analysis

Practical guidance is limited on developing core outcome sets for organizations rather than individuals such as clinicians or policy makers (Sinha et al., 2011). We will therefore look to consensus building frameworks and psychometrically-validated tools used in the clinical (Kirkham et al., 2017) and other data science research contexts for guidance (Board, 2019). Descriptive statistics–including median, mean, interquartile range and standard deviation—will benchmark consensus on the core outcomes set (von der Gracht, 2012) when there is >70% agreement on one rating, or 80% agreement across two contiguous ratings (Needham and de Loe, 1990). We will generate a core-outcomes set from those outcomes which are considered essential via panel consensus and which demonstrate low to no polarity based on IQRs less than 1 (Raskin, 1994; Rayens and Hahn, 2000).

### 2.3 Phase 3 validation of the stewardship maturity matrix tool

Borrowing from approaches used in the environmental impact assessment literature (Bockstaller and Girardin, 2003), two validation exercises will serve to test the tool’s “output” and “usability” among prospective end users.

#### 2.3.1 Phase 3 data collection

We will first develop hypothetical vignettes of stewardship practices that correspond to each of the five stewardship maturity levels outlined in the SMM and assign reference scores to them. Next, we will conduct cognitive interviews with prospective end users to validate how well user scores align with the reference (output validation). Cognitive interviewing is a specific approach to structured interviewing during which we will capture real-time feedback on user experience (Willis et al., 2004; Willis, 2005; Boeije and Willis, 2013). Interviewees ‘think aloud’ as they apply the SMM to assign an overall stewardship maturity score to each vignette until assessments reach a recommended interrater reliability score of 0.8 (Burla et al., 2008). Following the interviews participants will complete a System Usability Survey (Bangor et al., 2008; Lewis, 2018) to complement output validation data about the tool’s overall ease of use (user validation).

#### 2.3.2 Phase 3 participation selection

Interviewees will be purposively recruited from expert communities who have experience developing data management and release policies, standards and executable data access workflows in cloud environments.

#### 2.3.3 Phase 3 data analysis

We expect the validation exercises to generate quantitative as well as qualitative data. Both datasets will require their own analytical approaches. Pearson’s chi square test will enable us to compare reference scores with scores assigned by end users. User experience themes will also be synthesized from qualitative data emerging from the cognitive interviews using a content analysis approach. To enhance rigor, independent coders will develop an initial codebook from analyzing a sample of interview transcripts. Coders will then meet to resolve any discrepancies and revise the codebook as appropriate.

### 2.4 Pilot testing and implementation

Should we fail to reach interrater consensus during the cognitive interviews, or the usability tests reveal issues with internal validity, we will re-engage Delphi participants to further refine the SMM based on feedback from the validation studies. Upon successfully demonstrating the tool’s output validity and usability, we will pursue a pilot program with repository managers affiliated with cloud-native repositories. Pilot testing will inform the organizational factors to consider for implementation.

## 3 Limitations

The mixed-methods study design described in this protocol should be considered in light of several limitations and considerations. Delphi studies can be both time and resource intensive. It is possible that panelists are lost to attrition, which may skew the rating distributions. Second, engaging primarily institutional stakeholders to help develop the tool, may not adequately capture the perspectives and experiences of data contributors. Researchers could consider adapting the protocol in the future to solicit input directly from individuals who have previously shared their data, or plan to contribute their genomic data to cloud-native repositories in the future. Third, cloud computing and software engineering professionals skew largely white, European and male. Therefore, oversampling participants from groups commonly underrepresented in these technical fields, particularly during the Phase 2 validation phase, is critically important for promoting equity and representation as well as to ensuring external validity. Fourth, usability testing may not capture all relevant errors end users could make. Participants’ unfamiliarity with the concepts measured in Phase 2—for example ethics, stewardship and governance, time spent working in one’s role—as well as biases that can carry over from institutional environments are among the most common reasons why usability testing fails.

## 4 Conclusion and future directions

The development, validation, and implementation of an impact assessment tool is an important practical solution to a growing infrastructure problem for institutions that endeavor to track progress on genomic data stewardship in the cloud. This article outlines a mixed-methods protocol to rigorously develop and validate an assessment tool to monitor human genomic data stewardship in novel cloud environments. Research and development of a SMM for genomic data stewardship is especially timely as government investment in cloud-based data infrastructures expands (e.g., NIH STRIDES Initiative, https://cloud.nih.gov/about-strides/). Both institutional and public stakeholders benefit from transparent reporting of stewardship outcomes at the repository level. A reliable and usable SMM tool allows data managers, data access committee members, privacy officers, and other institutional officials to self-assess stewardship practices early and often. Scores generated from periodic assessment using the SMM tool could enable data stewards to identify ‘“quick wins” where higher ratings for some aspects require little effort to obtain” (Dunn et al., 2021). With the stewardship assessment criteria in mind, genomic researchers could proactively practice good stewardship when sharing or curating data they generate in their work. Researchers could also use stewardship scores to help guide their choices about which datasets to use for their projects. Finally, periodic assessment and routine reporting of stewardship outcomes using a standard SMM tool can improve repository practices in the long term while helping to sustain public trust in publicly funded genomic research in the future.

Future work will be needed to determine repository preparedness for implementing stewardship assessments as part of their annual reporting. Rigorous studies investigating the effects of transparent reporting of stewardship outcomes on more diverse data stakeholders (e.g., individual and community data contributors) are also needed. Cloud-native repositories could in the future seek certification for their commitment to responsible stewardship practice through programs sponsored under the CoreTrustSeal (https://www.coretrustseal.org/) and strike an advisory committee to review and assess new data infrastructure proposals. “If cloud technology is the future of biomedical science then, for genomics, the future is already here” (44). It is incumbent on data producers, users and regulators alike to prepare for this future in ways that are concordant with diverse value systems and as computer science and genomic data discovery evolve.

## Data Availability

The original contributions presented in the study are included in the article/Supplementary Material, further inquiries can be directed to the corresponding author.

## Abbreviations

ELSI—Ethical, legal, and social issues. FAIR—Findable, Accessible, Interoperable, Reusable. IQR—interquartile range. PaaS—Platform as a service. SMM—stewardship maturity matrix. SaaS—Software as a service.
