# Supplementary material for: A mixed-methods protocol to develop and validate a stewardship maturity matrix for human genomic data in the cloud
Source: Front Genet. 2022 Oct 14;13:876869. doi: 10.3389/fgene.2022.876869 (PMC9614211; doi:10.3389/fgene.2022.876869)
Supplement: Supplementary file 1 [file DataSheet1.docx]

**Supplementary Materials 1**. Example search strategy for literature review to refine a core outcomes list for stewardship outcomes

((("steward"[All Fields] OR "steward s"[All Fields] OR "stewarding"[All Fields] OR "stewards"[All Fields]) AND ("stewardship"[All Fields] OR "stewardships"[All Fields]) AND "steward*"[All Fields]) OR (("governability"[All Fields] OR "governable"[All Fields] OR "governance"[All Fields] OR "governances"[All Fields] OR "government"[MeSH Terms] OR "government"[All Fields] OR "governments"[All Fields] OR "government s"[All Fields]) AND "govern*"[All Fields])) AND ("human s"[All Fields] OR "humans"[MeSH Terms] OR "humans"[All Fields] OR "human"[All Fields]) AND ("genomic*"[All Fields] AND "genetic*"[All Fields] AND "genomics*"[All Fields] AND "genetics*"[All Fields]) AND ("ethic*"[All Fields] AND "bioethic*"[All Fields] AND ("biomedical"[All Fields] OR "biomedically"[All Fields]) AND "ethic*"[All Fields])
